# Specific Gut Microbiome and Serum Metabolome Changes in Lung Cancer Patients

**DOI:** 10.3389/fcimb.2021.725284

**Published:** 2021-08-30

**Authors:** Feng Zhao, Rui An, Liqian Wang, Jikang Shan, Xianjun Wang

**Affiliations:** ^1^Department of Laboratory Medicine, The Fourth School of Clinical Medicine, Zhejiang Chinese Medical University, Hangzhou, China; ^2^Department of Laboratory Medicine, Affiliated Hangzhou First People’s Hospital, Zhejiang University School of Medicine, Hangzhou, China; ^3^Department of Laboratory Medicine, Sir Run Run Shaw Hospital, Zhejiang University School of Medicine, Hangzhou, China

**Keywords:** lung cancer, gut, microbiota, serum metabolites, biomarkers

## Abstract

**Background:**

Lung cancer (LC) is one of the most aggressive, prevalent and fatal malignancies. Gut microbes and their associated metabolites are thought to cause and modulate LC development, albeit influenced by the host genetic make-up and environment. Herein, we identified and classified gut microbiota and serum metabolites associated with LC.

**Methods:**

Stool samples were collected from 41 LC patients and 40 healthy volunteers. The gut microbiota was analyzed using 16S rRNA gene sequencing. Serum samples were collected from the same LC patients (n=30) and healthy volunteers (n=30) and serum metabolites were analyzed using liquid chromatography-mass spectrometry (LC-MS). Microbiome and metabolome data were analyzed separately and integrated for combined analysis using various bioinformatics methods.

**Results:**

Serum metabolomics uncovered 870 metabolites regulated in 76 metabolic pathways in both groups. Microbial diversity analyses identified 15967 operational taxonomic units (OTUs) in groups. Of these, the abundance of 232 OTUs was significantly different between HC and LC groups. Also, serum levels of glycerophospholipids (LysoPE 18:3, LysoPC 14:0, LysoPC 18:3), Imidazopyrimidines (Hypoxanthine), AcylGlcADG 66:18; AcylGlcADG (22:6/22:6/22:6) and Acylcarnitine 11:0 were substantially different between HC and LC groups. Combined analysis correlated LC-associated microbes with metabolites, such as Erysipelotrichaceae_UCG_003, Clostridium and Synergistes with glycerophospholipids.

**Conclusions:**

There is an intricate relationship between gut microbiome and levels of several metabolites such as glycerophospholipids and imidazopyrimidines. Microbial-associated metabolites are potential diagnostic biomarkers and therapeutic targets for LC.

## Introduction

Lung cancer (LC) is one of the most prevalent and fatal malignancies globally. Data shows that, the incidence and deaths due to LC have been increasing yearly (Sung et al., 2021). Most LC patients are initially diagnosed when the disease is an advanced stage, hence such patients have a poor prognosis. Thus, early diagnosis can greatly improve disease management and the overall survival rates of LC patients. Identifying key biomarkers and mechanisms that promote development of LC can uncover powerful diagnostic and treatment targets for LC. However, these aspects are largely unexplored.

Accumulating evidence suggest that LC development is driven by a combination of genetic and environmental factors (Alexandrov et al., 2016; Malhotra et al., 2016). Recent studies have shown that the occurrence and development of LC is also related to human intestinal flora, where the interaction between these organisms influence functioning of several pathways such as metabolic, inflammatory and immune pathways (Dzutsev et al., 2015; Chen et al., 2017; Mao et al., 2018). Meanwhile, diet and physiological changes can affect the diversity and interaction between host microbial community (Shoaie et al., 2015; Song et al., 2015). The resilience and stability of the microbiome and its responsiveness to physiological, pathological and environmental changes make them and their associated metabolic pathways attractive diagnostic and treatment targets for numerous diseases (Magnusdottir et al., 2017; von Frieling et al., 2018).

Despite the progress in understanding the association of gut microbiome in LC patients, the profile and functional role of these organisms remain largely unknown (Hosgood et al., 2014; Qin et al., 2014; Rutten et al., 2014; Wang et al., 2014; Zhang et al., 2018; Gui et al., 2020; Zheng et al., 2020). In addition, several studies have demonstrated a strong relationship between gut microbiome as well as their metabolite and LC (DeBerardinis and Chandel, 2016; Pavlova and Thompson, 2016; Liu F et al., 2019; Song et al., 2020; Zheng et al., 2020). Gut microbiota can transform host nutrients into complex metabolites (Anand and Mande, 2018). The resultant metabolites play an important role in human health and can alter genotoxic or tumor suppressor functions through several mechanisms such as providing metabolic energy, promoting biosynthesis and modifying signaling proteins (Anand and Mande, 2018). Therefore, disruption of metabolite balance resulting from altered microbiome homeostasis may promote tumorigenesis.

However, little is known regarding interactions between gut microbiome and metabolites, and how they influence LC development. Studies in this area using conventional methods are limited by the high cost and restrictive nature of the invasive sample extraction procedures. Herein, we evaluated the respective microbial diversity and abundance of metabolites in fecal matter serum of LC patients and their association with the cancer.

## Materials and Methods

### Study Design

A total of 107 LC patients and 60 healthy individuals attending the Hangzhou First People’s Hospital between October 2019 and June 2020 were enrolled to this study. Overall, 81 individuals including 41 LC patients and 40 HC participants fulfilled the inclusion criteria and were therefore incorporated in the final study. The enrollment and selection process of the study participants is highlighted in **Supplementary Figure 1**. Demographic and clinical data of the study participants captured at baseline included body mass index (BMI), sex, age, medical history, family health history, lifestyle and dietary habits. For LC patients, other clinical and pathological features including tumor pathological type, tumor stage, serum squamous cell carcinoma antigen (SCC), neuron-specific enolase (NSE) and cytokeratin fragment (CYFRA21-1) were also captured. To be included in this study, participants met the following criteria: (1) ≥ 18 but < 80 years old; (2) have been histopathologically confirmed with lung cancer and had no history of malignancy. LC patients (1) with history of chemotherapy, radiotherapy or cancer surgery; (2) with other underling malignant tumors; (3) with cardiovascular diseases (myocardial infarction or stroke); (4) have received probiotics, antibiotics, proton pump inhibitors (PPI), and hormone drugs within the past 2 months before enrollment; (5) with history of gastrointestinal surgery; (6) with inflammatory bowel disease (IBD) and irritable bowel syndrome (IBS); (7) with diabetes and depression were excluded from the study. All healthy participants had to have normal bowel habits. Also, both groups must not have used antibiotics, probiotics, prebiotics or synbiotics within the two months prior to sampling. The protocol for this study was proved by the Clinical Research Ethics Committee of Hangzhou First People’s Hospital.

### Sample Collection

Stool and serum samples were collected in the morning after overnight fasting (≥ 8h). The stool samples were divided into 5 equal parts (each 200mg), put in sterile frozen pipes and in an ice box and transported immediately to the laboratory for storage at -80°C. Blood samples were collected in coagulant tubes. The tubes were gently shaken after blood collection and centrifuged at 3000r for 10 minutes at room temperature. The supernatant (serum) was collected in 1.5ml frozen tubes and stored at -80°C pending further analyses.

### Microbial DNA Extraction in Fecal Matter

The genomic bacterial DNA in stools was extracted using the E.Z.N.A.^®^ Stool DNA Kit (Omega, USA), according to the manufacturer’s protocols. The integrity and fragment sizes of the extracted DNA were analyzed using 1% agarose gel electrophoresis. The DNA was quantified using NanoDrop 2000 (Boston, USA).

### High-Throughput 16S Ribosomal RNA Gene Sequencing

The V3-V4 variable region of 16S rRNA was amplified using 341F: 5’-CCTACGGGNGGCWGCAG-3’ and 805R: 5’- GACTACHVGGGTATCTAATCC -3’ primers and Phusion^®^ Hot Start Flex 2X Master Mix (New England biolabs, USA). The 5’ ends of the primers for each sample were tagged with specific barcodes. The amplicons were purified using AMPure XT beads (Beckman Coulter Genomics, USA) and thereafter quantified using Qubit (Invitrogen, USA). The amplicons were processed, sequenced and assessed for size and quantity using the Agilent 2100 Bioanalyzer (Agilent, USA). The amplicon Library was then quantified using the Quantification Kit for Illumina (Kapa Biosciences, USA). Sequencing was performed using the NovaSeq PE250 platform (Illumina, USA), according to the manufacturer’s protocol.

### Analysis of Sequence Data

The paired-end reads were assigned to samples based on their unique barcodes, before cutting off the barcodes and primers. Paired end reads were merged using the FLASH software. The raw reads were cleaned using fqtrim (v. 0.94). Chimeric sequences were filtered using the Vsearch software (v2.3.4). Dereplication was performed using DADA2 to obtained feature table and sequence. Alpha and beta diversities were calculated using QIIME2, whereas the corresponding phylogenetic tree was constructed using R software V. 3.5.2. Alignment of sequences and annotation of species was performed using Blast tool, whereas alignment of sequences was performed using SILVA and NT-16S.

### Processing Samples and Analysis of Serum Samples

After thawing on ice, metabolites in the serum samples were extracted using 50% methanol Buffer. Briefly, 120 μL of precooled 50% methanol was added to 20 μL of sample, vortexed for 1 minute, incubated at room temperature for 10 minutes and thereafter at -20°C, overnight. After centrifugation at 4,000 g for 20 minutes, the supernatants were transferred into new 96-well plates. QC samples were prepared by pooling together 10 μL of each extract. The metabolites were stored at -80°C prior to the Liquid Chromatography-Mass Spectrometer (LC-MS) analysis.

### LC-MS Analysis

The samples were analyzed using a TripleTOF 5600 Plus high-resolution tandem mass spectrometer (Boston, USA) with both positive and negative ion modes. Chromatographic separation was performed using an ultra-performance liquid chromatography (UPLC) system (Boston, USA). Reversed-phase separation was performed using an ACQUITY UPLC T3 column (100mm*2.1mm, 1.8μm) (Boston, USA). Eluted metabolites were detected and quantified using the TripleTOF 5600 Plus system. For the positive-ion mode, the ion spray floating voltage was set at 5kV, whereas for the negative-ion mode, the voltage was set at ‐4.5kV. The MS data was acquired in IDA mode. The TOF mass range was 60-1200Da. During the entire period, the mass accuracy was calibrated after every 20 samples. Furthermore, the QC sample was analyzed after every 10 samples to evaluate the stability of the LC-MS.

### Metabolomics Analysis

Processing of the MS data including peak picking, peak grouping, retention time correction, second peak grouping and annotation of isotopes and adducts was performed using XCMS software. LC-MS raw data files were converted into mzXML format before processing using XCMS, CAMERA and metaX toolbox in R software. Each ion was identified by combining retention time (RT) and m/z data. The intensity of each peak was recorded. A three-dimensional matrix of arbitrarily assigned peak indices (retention time‐m/z pairs), sample names (observations) and ion intensities (variables) was also generated.

The metabolites were annotated using online KEGG and HMDB databases by matching molecular mass data (m/z) of samples with those in the database. If the difference between observed and the database mass was less than 10 ppm, the metabolite would be annotated with its molecular formula further identified and validated by isotopic distribution measurements. The identity of the metabolite would further be validated using an in‐house fragment spectrum library of metabolites. Peak intensity data was further preprocessed using metaX. Peaks in less than 50% of QC samples or 80% of biological samples were removed, whereas those with missing values were imputed with the k‐nearest neighbor algorithm to further improve the data quality. Identification of outliers and evaluation of batch effects were performed using PCA based on the pre‐processed dataset. Quality control‐based robust LOESS signal correction was fitted to the QC data with respect to the order of injection to minimize drifting of signal intensity over time. In addition, the relative standard deviations of the metabolic features were calculated across all QC samples, with those > 30% removed.

### Statistical Analysis

Continuous variables were expressed as mean ± standard deviation (SD). Comparison between two groups was performed using Student’s t test or separate variance estimation t-test for independent samples. Differences between categorical variables were assessed using chi-square test. The relationship between metabolites or between species and metabolites were assessed using Spearman’s rank correlation analysis. Moreover, the association of systemic inflammatory markers with gut butyrate-producing bacteria were assessed using Spearman’s rank correlation. For LC-MS/MS analysis, Supervised PLS‐DA was performed using metaX to discriminate different variables between groups. The VIP threshold for important features was set at1.0. Statistical significance was set at P < 0.05. Raw P values were adjusted for multiple tests using an FDR (Benjamini–Hochberg). Data were analyzed using SPSS version 22.0 (Statistical Product and Service Solutions, IBM, USA) and R version 3.5.2 (R Foundation for Statistical Computing, Austria).

## Results

### Population and Clinical Characteristics

Herein, 16S rRNA gene sequencing of microorganism in fecal matter and LC-MS analysis of serum samples were performed to investigate the differences in gut microbiome and metabolites between LC patients and healthy individuals. Overall, two LCs (LC_13, LC_34) were excluded because they lacked pre-treatment serum samples, so we analyzed samples for 79 individuals (39 LCs and 40 HCs). For the metabolome samples, four samples (HC_33, LC_16, LC_17 and LC_18) were excluded according to PCA (principal component analysis) because they deviated significantly from the major groups (**Supplementary Figure 2**). Therefore, only data for 56 individuals (27 LCs and 29 HCs) was included in the final 16S rRNA, metabolomic and correlation analyses. All participants were Han Chinese from Zhejiang region. There were no significant differences in age, sex and BMI, but several clinical parameters including white blood cell (WBC), lymphocyte (L), platelet-lymphocyte ratio (PLR), prognostic nutritional index (PNI), total bilirubin (TBIL), triglyceride (TG) levels among others, were differed between the HC and LC group. Details of demographic and clinical characteristics of the LC and HC participants are shown in **Supplementary Table 1**.

### Gut Microbial Profiles

After filtration, we obtained 4,074,514 high-quality sequences from the 79 samples, averaging 51,576 sequences per sample. There were also 15,967 OTUs, averaging 202 OTUs per sample (**Supplementary Table 2**). The rarefaction curve of richness and diversity (observed OTUs, chao, shannon, simpson index) in the two groups tended to be flat or reached a plateau, demonstrating satisfactory sequencing depth (**Supplementary Figure 3**).

**Figure 1:**
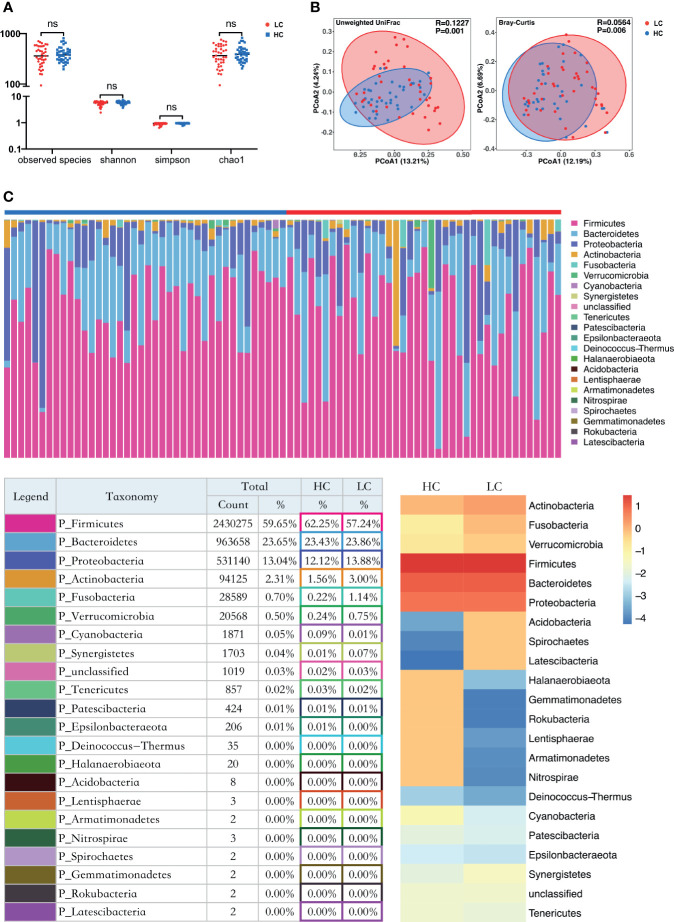
Structure and diversity analysis of the gut microbiota. **(A)** Differences in alpha diversity between LC and HC based on the observed species, chao1, shannon, and simpson indices. LC, lung cancer group; HC, healthy control group; NS, not significant. **(B)** Beta diversity differences between the LC and HC were estimated by Principle coordinates analysis (PCoA). Left, Unweighted UniFrac; right, Bray-Curtis. LC group (red dots); HC group (blue dots). The percentage of variance explained by the first two principal coordinates (PCs) is labeled in brackets. **(C)** The proportions at the bacteria phylum level for each group. n = 39 for the LC group, and n = 40 for the HC group. Bottom left: The relative proportion of dominant taxa at the phylum level was assessed by the assignment of microbial taxa, with the most dominant phyla being the Firmicutes, Bacteroidetes, Proteobacteria and Actinobacteria. Bottom right: Heat map showing the relative abundance of the 22 phyla in the two sample groups. The phyla are showed in the rows and the relative abundance is indicated by a color gradient.

Alpha diversity analysis revealed there was no significant difference in Sobs, Chao, Shannon and simpson index between the LC and HC groups (**Figure 1A**). However, Principle coordinate analysis (PCoA) and Analysis of similarities (ANOSIM) test for Beta diversity revealed a significant difference in the composition and abundance of gut microbiota between the two groups (Unweighted Unifrac P = 0.001 and Bray-Curtis P = 0.006) (**Figure 1B**).

### LC-Related Changes in the Composition of Gut Microflora

Taxon-dependent analysis (**Figure 1C**) revealed 22 phyla in each of the LC and HC group, with Firmicutes, Bacteroidetes, Proteobacteria and Actinobacteria being the most dominant phyla. Firmicutes was the most predominant phylum, accounting for 62.25% and 57.24% of gut microbiota in the HC and LC group, respectively. Additionally, Actinobacteria were more predominant in LC (3%) than HC group (1.56%). A similar trend was observed for Fusobacteria (0.22% for HC and 1.14% for LC). Cyanobacteria were more abundant in HC (0.09%) than LC group (0.01%). There was no association between Firmicutes/Bacteroidetes and LC (**Supplementary Figure 3F**), Although it has been considered that Firmicutes/Bacteroidetes ratio is associated with a variety of diseases (Magne et al., 2020).

**Figure 2:**
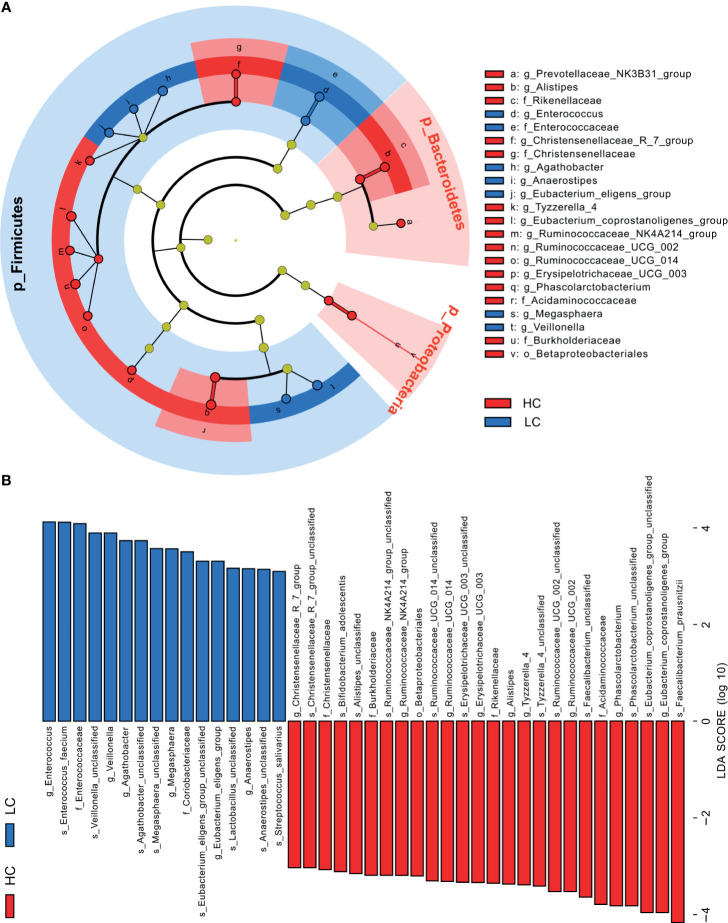
Linear discriminant analysis (LDA) combined with effect size (LEfSe). **(A)** Cladogram showing the phylogenetic distribution of microbiota associated with group HC or LC. **(B)** Histogram of the LDA scores, where the LDA score indicates the effective size and ranking of each differentially abundant taxon (LDA > 3).

**Figure 3:**
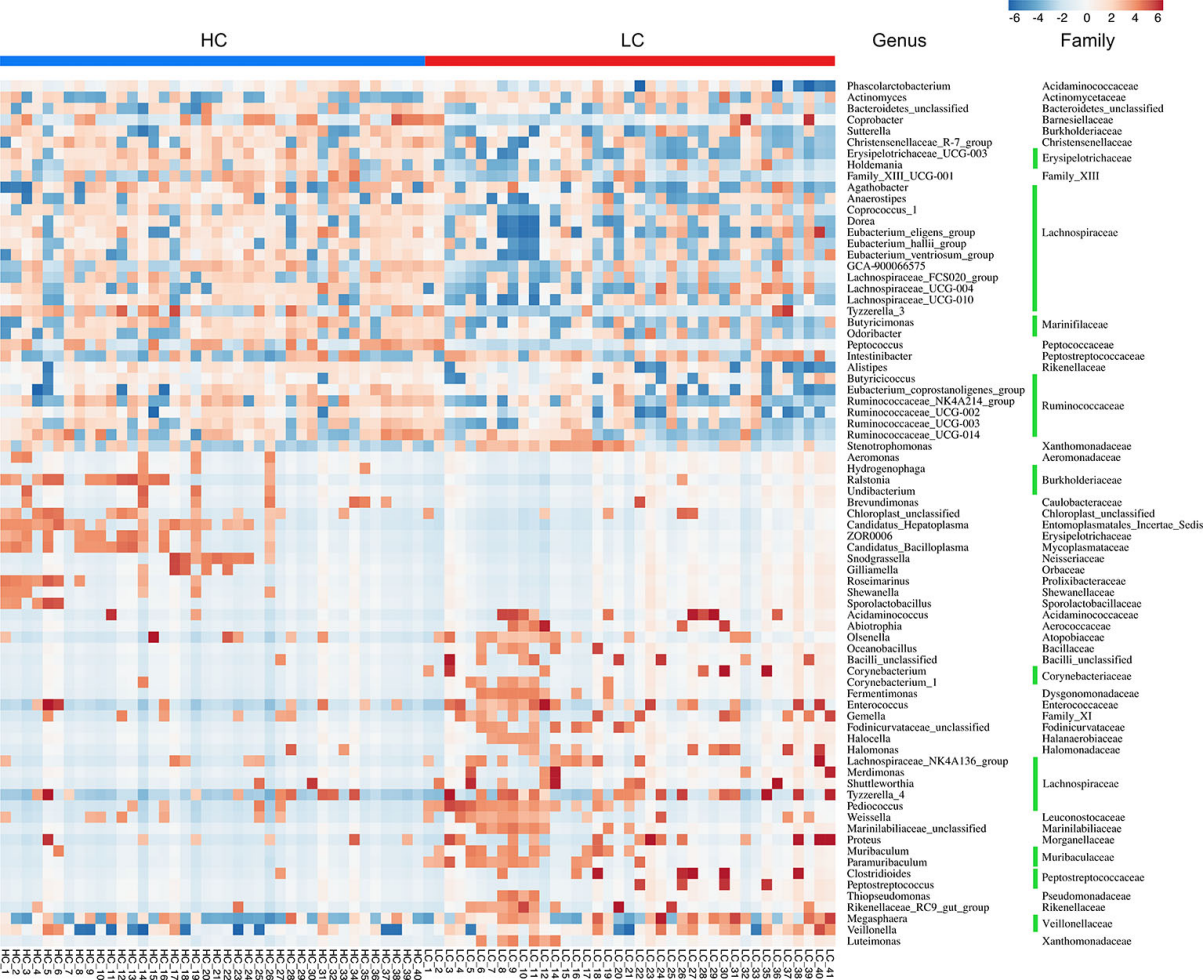
Heat map illustrating relative abundance of the 77 OTUs that differentiated the HC and LC groups. OTUs z-transformation data from low (in blue) to high (in red) abundance. Data were compared by Wilcoxon rank sum test (Mann-Whitney U test). All 77 OTUs were distributed to families and genera.

Further analyses revealed that at phylum level, Tenericutes (P < 0.0001) and Cyanobacteria (P = 0.0183) were significantly more abundant in HC group, whereas Halanaerobiaeota (P = 0.0202) were more abundant in LC group (**Supplementary Figure 4A**). At Genus level, members of 77 genera were significantly different between LC and HC groups. Among them, Actinomyces (P < 0.0051), Veillonella (P = 0.0057), Megasphaera (P = 0.0149), Enterococcus (P = 0.0183) and Clostridioides (P = 0.0202) were more abundant in LC than in HC group (**Supplementary Figure 4B**). Because discriminative analysis did not identify major taxa differences, we used LDA Effect Size (LEfSe) analysis to generate a Cladogram to reveal differences in taxa abundance between LC and HC (**Figure 2**). We found significant differences in 42 OTUs (LDA>3), in which Enterococcus, Veillonella, Agathobacter, Megasphaera and Coriobacteriaceae (all LDA scores (log10)>3.5) were more abundant in the LC group, relative to the HC group. Contrarily, Faecalibacterium, Eubacterium_coprostanoligenes_group, Phascolarctobacterium, Acidaminococcaceae and Ruminococcaceae_UCG_002 were significantly more abundant in the HC (all LDA scores (log10) > 3.5) than LC group. The heat map for the relative abundance of the 77 genera is shown in **Figure 3**. In general, 42 OTUs were more abundant in the HC group, compared with 35 OTUs in the LC group. (**Supplementary Table 3**). Overall, these findings demonstrated that the abundance of microbes in the LC group was relatively lower than those in the HC group, sufficient enough to distinguish healthy individuals from LC patients.

Next, we performed liquid chromatography-mass spectrometry (LC-MS) of serum samples from 27 LC patients and 29 healthy individuals. Firstly, we used Venn diagram to determine whether the intestinal flora profile (n = 27 and n = 29, LC *vs*. HC) could represent the overall status (n =39 and n = 40, LC *vs*. HC). Comparing the microbial profiles of LC patients and HC participants (n = 39 and n = 40, LC *vs*. HC) showed that the common OTUs was 12527 (78.5%, **Supplementary Figure 5A**), while the common altered genera was 33 (42.9%, **Supplementary Figure 5C**; P<0.01). Thus, the flora profile in the 27 LC patients and 29 healthy individuals was a good representative of the overall intestinal flora (LC=39 and HC=40, LC *vs*. HC).

### Overall Blood Metabolome of LC and HC Groups

Considering the large impact of the gut microbiome on blood metabolites (Wikoff et al., 2009) and the above findings, it was hypothesized that gut microbes in the LC patients impact on blood metabolic pathways. Accordingly, non-targeted metabolomics based on LC-MS identified and quantified 870 metabolites in the HC and LC groups (**Supplementary Table 5**). KEGG analysis revealed that synthesis of blood metabolites was regulated by 76 different metabolic pathways, including Alpha Linolenic Acid and Linoleic Acid Metabolism pathway (7 metabolites), Urea Cycle pathway (8) and Phenylacetate Metabolism pathway (3) among others (**Supplementary Figure 6A**).

### Group Differential Blood Metabolites

**Figure 4:**
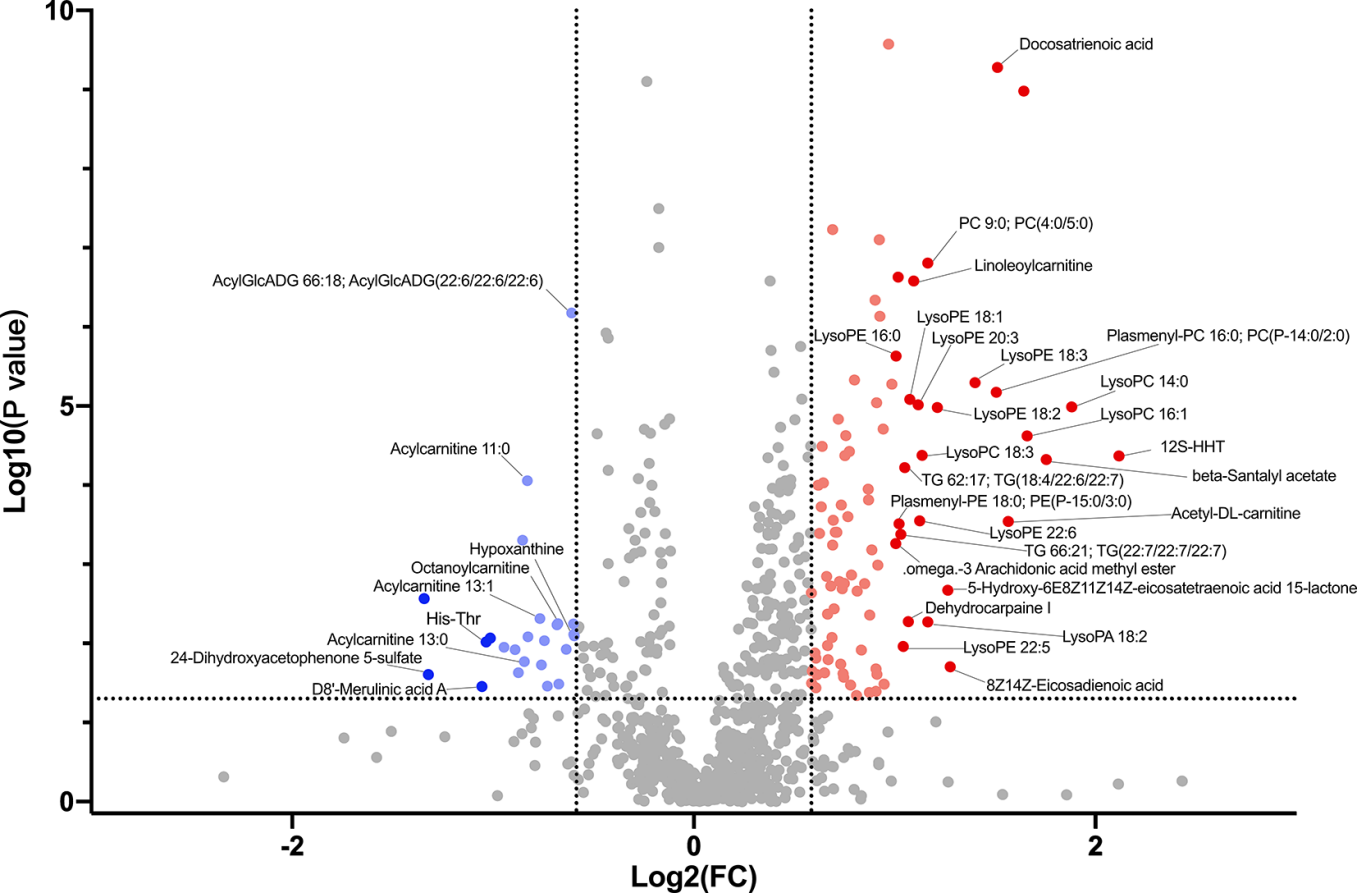
Volcano plot showing accumulated [log2 (FC) on X axis] metabolites that were significantly different [log10 (P value) on Y axis] between LC group and HC group.

**Supplementary Table 5** shows major metabolites at substantially different levels in the HC and LC groups. The most abundant metabolites in the HC group mainly included organooxygen compounds (2,4-Dihydroxyacetophenone 5-sulfate), benzene and substituted derivatives (D8’-Merulinic acid A, 1,2,3-Trihydroxybenzene), carboxylic acids and their derivatives (His-Thr), imidazopyrimidines (Theobromine, ParaXanthine, Hypoxanthine) and large fatty acyls (Acylcarnitine 13:0, Acylcarnitine 11:0, Acylcarnitine 13:1, Octanoylcarnitine). In contrast, the LC group displayed higher levels of fatty acyls (12S-HHT, Acetyl-DL-carnitine, Docosatrienoic acid), glycerophospholipids (LysoPC 14:0, LysoPC 16:1, Plasmenyl-PC 16:0; PC(P-14:0/2:0), LysoPE 18:3) and prenol lipids (beta-Santalyl acetate). These findings suggest that LC group had higher lipid metabolism and oxidation relative to the HC group (**Supplementary Table 6** and **Figure 4**).

### Multiple Analytical Approaches Revealed the Discriminatory Metabolites Between the HC and LC Groups

**Figure 5:**
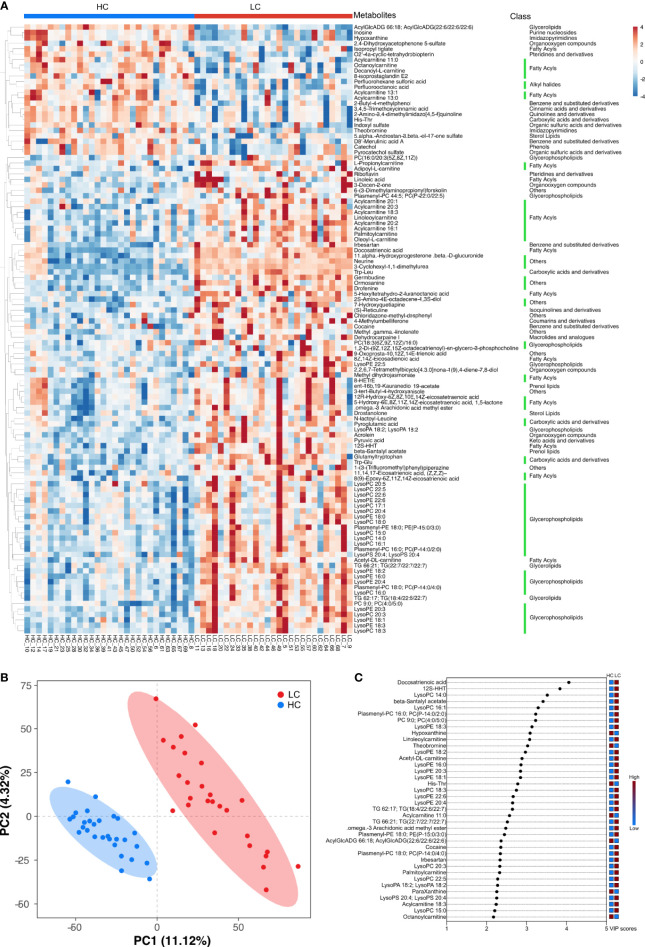
Key discriminatory metabolites were identified by clustering and multivariate correlation analysis between HC group and LC group. **(A)** Hierarchical clustering analyses (HCA) was based on the relative abundance of normalized numbers of metabolomics data from HC and LC group. **(B)** OPLS-DA scores displaying the discrimination between HC group and LC group by the first two principal components (PCs). **(C)** Important discriminatory metabolites displayed on variable importance in projection (VIP) plot obtained from OPLS-DA.

Hierarchical clustering (HCA) analysis revealed higher levels of serum organoheterocyclic compounds and benzenoids in the HC group relative to the LC group. In contrast, serum lipids and lipid-like molecules, organic acids and their derivatives, organic oxygen compounds were significantly higher in the LC group than in the HC group (**Figure 5A**). Correlation analysis further revealed a strong association between the above metabolites and with LC phenotype (**Supplementary Figure 6B** and **Supplementary Table 7**). More specifically, we observed higher levels of Imidazopyrimidines such as Hypoxanthine, Theobromine and ParaXanthine, Benzene and their derivatives such as 1,2,3-Trihydroxybenzene and D8’-Merulinic acid A as well as Fatty Acyls such as Octanoylcarnitine, Acylcarnitine 11:0/13:0/13:1 in the HC group. Contrarily, Glycerophospholipids such as LysoPC 14:0, LysoPC 16:1, Plasmenyl-PC 16:0 PC (P-14:0/2:0), LysoPE 18:3, LysoPE 18:2, LysoPA 18:2 and PC 9:0, PC (4:0/5:0), Fatty Acyls such as 12S-HHT, Acetyl-DL-carnitine, Docosatrienoic acid, Acylcarnitine 18:3/20:1/20:2, 8Zand 14Z-Eicosadienoic acid were more abundant in the LC group. PCA revealed comparable findings, which based on the first two principal components, PC1 (11.12%) and PC2 (4.32%) (**Figure 5B**). OPLS-DA analysis also revealed consistent findings, in which the level of several metabolites including Docosatrienoic acid, 12S-HHT, LysoPC 14:0, beta-Santalyl acetate, LysoPC 16:1, Plasmenyl-PC 16:0;PC(P-14:0/2:0), PC 9:0;PC(4:0/5:0), LysoPE 18:3, Hypoxanthine, Linoleoylcarnitine, Theobromine, LysoPE 18:2, Acetyl-DL-carnitine, LysoPE 16:0, LysoPE 20:3 and LysoPE 18:1 was significantly different between LC patients and healthy individuals (**Figure 5C**).

### Multi-Omics Approach Reveals Differences Between HC and LC Groups

**Figure 6:**
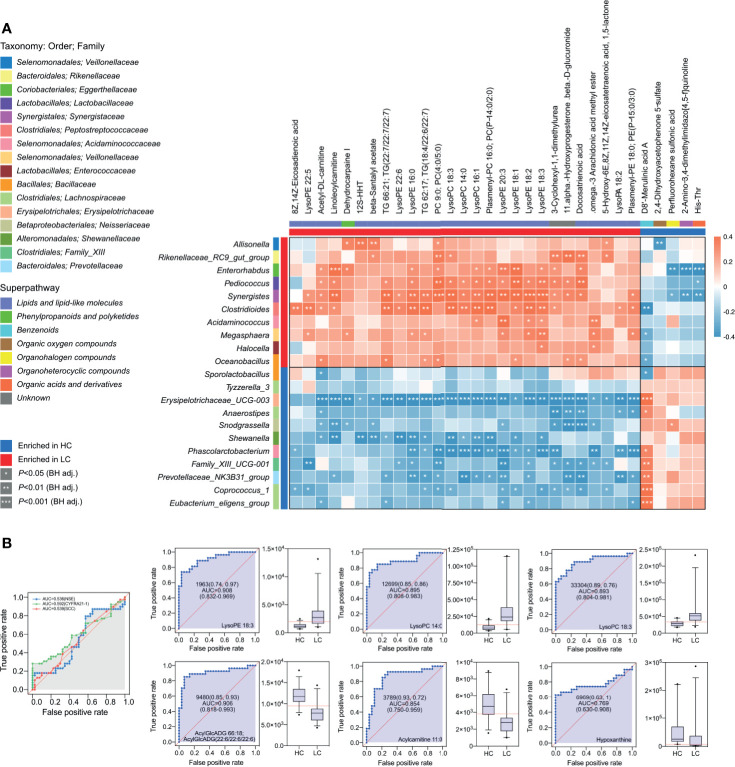
Integrated correlation analysis of microbes and metabolites. Heatmap of Spearman’s rank correlation analysis in the HC group and LC group. **(A)** The Enrichment is indicated by colored bars on the left and top of the plot in either group. Red, positive correlation; blue, negative correlation. Significant correlations regions are denoted by white stars (*P-value < 0.05; **P-value < 0.01; ***P-value < 0.001). **(B)** Metabolite abundance biomarker analysis showed high AUCs values for NSE, CYFRA21-1, SCC, LysoPE 18:3, LysoPC 14:0, LysoPC 18:3, AcylGlcADG 66:18; AcylGlcADG (22:6/22:6/22:6), Acylcarnitine 11:0, Hypoxanthine.

**Figure 7:**
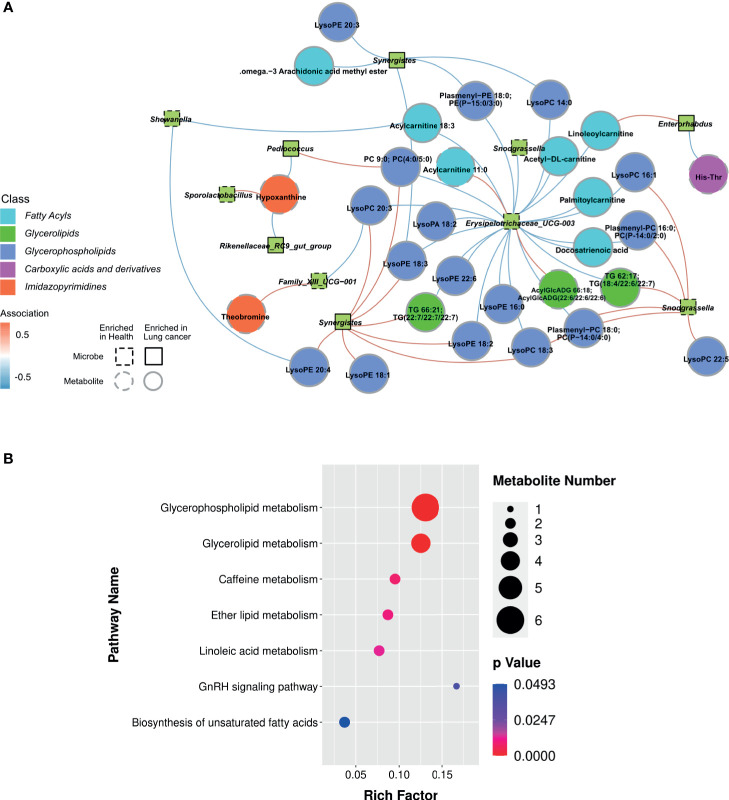
Lung cancer-associated networks based on integrated fecal microbiome and serum metabolome. **(A)** Mapping of association networks for correlations between bacterial species and metabolites using integrated microbiome and metabolome datasets. Red connecting lines indicate positive correlation between nodes, whereas blue lines indicate negative correlations. Bacterial species and metabolites enriched in samples from healthy individuals and those with lung cancer are indicated by dashed lines or solid lines, respectively. Black borders indicate significantly different strains and gray borders indicate significantly different metabolites (Spearman’s rank correlation analysis, r > 0.4, P < 0.05). **(B)** KEGG enrichment scatter plots show significant alterations in the biological processes and metabolism in the serum of lung cancer patients.

Based on the above findings, we assessed the relationship between 21 genera and 28 metabolites in LC (**Supplementary Table 8**). A strong positive correlation was observed between the abundance of several microbial genera and level of serum metabolite in LC group (**Figure 6A**). Network analysis based on the integrated metabolomic and genomic datasets was conducted to identify broader association between the microbiome and LC-related metabolites (**Figure 7A**). In this network diagram, a microbiome/metabolite cluster is defined. The associations between g:Erysipelotrichaceae_UCG-003, g:Phascolarcto-bacterium, g:Clostridioides, g:Synergistes and 27 metabolites are indicated. Thus, this cluster represents a short list of species and metabolites associated with the disease for future testing in clinical models. KEGG analysis of data in the MetPA database (part of MetaboAnalyst) (www.metaboanalyst.ca) revealed the unique metabolic pathways between the LC and HC group (**Figure 7B**). Particularly, Glycerophospholipid, Glycerolipid, Caffeine, Ether lipid and Linoleic acid metabolism pathways as well as GnRH signaling and unsaturated fatty acids biosynthesis pathways are the main pathways in LC group.

Based on the coefficient of variation (CV) for the abundance of serum metabolites, we selected a CV of 0-0.25 (less noise, better as a biomarker) (**Supplementary Table 6**). Metabolites with the lowest CVs (least variability) in LC group included LysoPC 20:3, Plasmenyl-PC 18:0; PC(P-14:0/4:0), LysoPC 18:0, 12S-HHT, LysoPC 16:1, LysoPE 18:0, LysoPA 18:2, LysoPC 20:4, LysoPE 18:3, LysoPE 16:0 and LysoPE 18:1, whereas those with higher CVs (greater variability) included PC(18:2(9Z,12Z)/18:2(9Z,12Z)), Acylcarnitine 20:3, Stearamide, PC(16:0/20:3(5Z,8Z,11Z)) and Plasmenyl-PE 18:0 and PE(P-15:0/3:0). For HC group, metabolites with the lowest CV included Acylcarnitine 13:1, Hypoxanthine, AcylGlcADG 66:18; AcylGlcADG (22:6/22:6/22:6), 1,2,3-Trihydroxybenzene and His-Thr, whereas those with higher CV included 2,4-Dihydroxyacetophenone 5-sulfate, Octanoylcarnitine, D8’-Merulinic acid A and Theobromine.

Considering the high abundance of glycerophospholipids in LC group, further analyses were conducted on them. Area under the receiver operating characteristic (ROC) curve for the association between specific glycerophospholipids and LC was as follows LysoPE 18:3 (AUC, 0.908), LysoPC 14:0 (AUC, 0.895), LysoPC 18:3 (AUC, 0.893), AcylGlcADG 66:18; AcylGlcADG(22:6/22:6/22:6) (AUC, 0.906), Acylcarnitine 11:0 (AUC, 0.854) and Hypoxanthine (AUC, 0.769) all at P<0.001 (**Figure 6B**). Thus, the diagnostic potential of glycerophospholipids for LC was superior to that SCC (AUC, 0.539; P = 0.56), NSE (AUC, 0.536; P = 0.58) and CYFRA21-1 (AUC, 0.592; P = 0.16).

## Discussion

Gut microbiota are all microorganisms that live in the digestive tract (Gilbert et al., 2018). Even though the total number of gut microbiota equals that of human cells, gene expression of these organisms is more than 150 times that in human cells (Human Microbiome Project, C 2012). Gut microbiotas perform numerous essential functions in the human gut such as fermentation of food components into absorbable metabolites. The resultant metabolites in turn regulate numerous pathways related to energy balance, nutrient intake and immune homeostasis (Kamada et al., 2013; Poutahidis and Erdman, 2016; Heintz-Buschart and Wilmes, 2018; Mithieux, 2018). Meanwhile, increasing evidence has linked microbiome and their metabolome to lung carcinogenesis (Kumar et al., 2017; Zheng et al., 2020). Moreover, the gut microbiome substantially influences the level of blood metabolites (Marcobal et al., 2013; Wilmanski et al., 2019; Lee-Sarwar et al., 2020). Therefore, analysis of gut microbiome and serum metabolome can potentially be used for cancer diagnosis. Advances in high-throughput tools have revolutionized genetic and molecular research and have led to discovery of numerous disease diagnostic biomarkers and uncovered other highly complex interactions in organisms. Compared to fecal metabolomics, serum metabolomics better reflects the interactions between the intestinal flora and distal organs and pathways (Wilmanski et al., 2019; Lee-Sarwar et al., 2020). Therefore, serum metabolomics can potentially identify diagnostic biomarkers. Herein, we employed this approach to explore the association between gut flora and metabolic pathways associated with lung cancer.

In the present study, LC patients displayed significantly high levels of serum metabolites such as Fatty Acyls (Docosatrienoic acid, 12S-HHT, Linoleoylcarnitine, Acylcarnitine 18:3/20:1/20:2, etc.) and Glycerophospholipids (LysoPC 14:0, LysoPC 16:1, Plasmenyl-PC 16:0; PC(P-14:0/2:0), PC 9:0; PC(4:0/5:0), LysoPE 18:3, etc.). High serum phospholipids, lysophospholipids and fatty acids in LC patients have been previously reported (Ros-Mazurczyk et al., 2017; Yu et al., 2017; Zhang et al., 2020). Lipids, especially phospholipids, participate in cellular trans-membrane transport, energy metabolism, signal transduction and cancer development (Pendaries et al., 2003; Ogretmen and Hannun, 2004; Gorke et al., 2010; Lee et al., 2012; Santos and Schulze, 2012). In our study, we also observed high serum LysoPA 18:2 levels in LC group, relative to HC group. Lysophosphatidic acid (LPA) is one of the active components of lysophospholipids and regulates transmission of extracellular signals and functioning of intracellular second messengers (Lee et al., 2020). As early as 1991, Merchant et al. identified lysophospholipid components such as LPA in malignant tumor tissues (Merchant et al., 1991). Later in 2003, Gordon Mills reported that LPA levels positively correlated with tumorigenesis, invasion and metastasis of cancers (Mills and Moolenaar, 2003). Recent related studies further demonstrated that LPA mainly inhibits apoptosis of tumor cells, thus promotes proliferation of these cells in situ. This promotes tumor angiogenesis, adhesion and migration of tumor cells, leading to the formation of cancer emboli (Tsujiuchi et al., 2014; Yung et al., 2014; Valdes-Rives and Gonzalez-Arenas, 2017; Benesch et al., 2018; Tigyi et al., 2019; Xu, 2019). LPA production is regulated by two main pathways. In the main pathway, phospholipase A (PLA1 or PLA2) catalyzes the production of lysophospholipids (LP), which is converted to LPA by prolidase (PLD) (Ye, 2008). Alternatively, autotoxin (ATX) present in tumor cells, fibroblasts and vascular smooth muscle cleaves the main groups in LP (choline, ethanolamine or serine), transforming it into LPA (Aoki et al., 2008). Interestingly, LysoPC 14:0/16:1/18:3 and LysoPE 16:0/18:1/18:2/18:3/20:3/22:5/22:6 which are highly expressed in the LC cells relative to normal cells can be converted to LPA by ATX, where they drive cancer processes. Serum AcylGlcADG 66:18;AcylGlcADG(22:6), Acylcarnitine 11:0/13:0/13:1, Octanoylcarnitine, Hypoxanthine and ParaXanthine levels were also high in HC group. However, research shows that these metabolites are significantly low in the serum of patients with different cancers (Long et al., 2017; Kim et al., 2019; Park et al., 2019; Zoni et al., 2019). Acylcarnitine is a key metabolite in cellular metabolism. Binding of acylcarnitine to fatty acids activates β-oxidation of the fatty acids in the mitochondria (Brosnan and Brosnan, 2009). Translocation of long-chain acylcarnitines across the mitochondrial matrix requires specific transferases such as carnitine/acetylcarnitine translocase (CACT), carnitine palmitoyl transferase (CPT). Contrarily, acylcarnitines with medium-chain fatty acids directly move through the mitochondrial membrane, where they fuel energy production (Li et al., 2019). In cancer cells, acylcarnitine metabolism participates in regulating switch between glucose and fatty acid metabolism. As such, is it precisely triggers metabolic flexibility in cancer cells (Melone et al., 2018). Levels of different chain lengths acylcarnitines in cancer cells are regulated by metabolic reprogramming in the cancer cells (Wang et al., 2018). This balances energy production and consumption as well as the synthesis of metabolic intermediates that drive cancer processes (Fujiwara et al., 2018). Abnormal expression of enzymes involved in acylcarnitine metabolism may lead to the accumulation of acylcarnitines with specific chain lengths (Hagenbuchner et al., 2018). For example, in prostate cancer cells, reduced expression of CPT and CACT negatively affects the oxidation of fatty acids (Valentino et al., 2017). In addition, significant alterations in the carnitine/acetylcarnitine pathway have been found in patients with bladder cancer. In patients with non-muscle-invasive bladder cancer, CPT and CACT expression are significantly downregulated compared to normal bladder tissue (Kim et al., 2016). the lack of CPT and CACT expression resulted in the accumulation of long-chain acylcarnitines and reduced level of short- and medium-chain acylcarnitines in the circulation (Kim et al., 2016; Valentino et al., 2017). We speculate this result from the high catabolism of medium-chain acylcarnitine, following the shift in energy metabolism in lung cancer cells. This may explain the lower levels of medium-chain acylcarnitine in the LC but higher levels of long-chain acylcarnitine in the LC group. Furthermore, xanthine and hypoxanthine are mostly under –expressed in cancer tissues and polyps, which may result from high DNA synthesis (adenine utilization) in the hyperproliferative tissues (Long et al., 2017). Patients with non-Hodgkin’s lymphoma have substantially low levels of urine hypoxanthine, relative to healthy individuals (Yoo et al., 2010). Also, several studies have demonstrated low serum hypoxanthine levels in patients with gastric and colorectal cancers as well as glioblastoma cancers (Jung et al., 2014; Kim et al., 2015; Bjorkblom et al., 2016). Similarly, in this study, we found significantly low serum hypoxanthine in LC patients, relative to HC individuals. Overall, we found clear and significant difference in serum metabolites between LC patients and healthy individuals.

Changes in the abundance of gut flora are a common hallmark of neoplastic disease (Schwabe and Jobin, 2013; Garrett, 2015). Intestinal flora such as *Fusobacterium nucleatum, Escherichia coli, Bacteroides fragilis* and *Aspergillus* have been associated with carcinogenesis (Schwabe and Jobin, 2013; Garrett, 2015). However, differences in microbiota may not be used to clearly explain the role of microbiota in health and disease (Integrative, H.M.P.R.N.C 2014). Therefore, the use of a prospective multi-omics approach combined with a comprehensive analysis of microbes as well as metabolites, may be one way to unravel the pathogenesis of the disease. During the multi-omics analysis, we need to avoid the influence of environmental factors such as diet, antibodies and other drugs, and hygiene on microbiomics and metabolomics results. Meanwhile, we also need to overcome the common challenges in multi-omics analysis, such as customized and sophisticated software, integrated data repositories and standardized sampling of blood, colon biopsy and stool (Panagi et al., 2019). In this study, we found that the abundance of Erysipelotrichaceae_UCG_003 and Phascolarctobacterium were substantially more abundant in HC group than LC group. Erysipelotrichaceae_UCG_003 is one of the main butyrate producing bacteria (Liu S et al., 2019), whereas Phascolarctobacterium participates in the synthesis of short-chain fatty acids (SCFAs) (Zhang et al., 2015). Propionate in a SCFA that modulates the immune system and proliferation of cancer cells (Sivaprakasam et al., 2016), hence maintain intestinal tract homeostasis (Liu et al., 2018), inhibits histone deacetylase and performs several anticancer functions (Conte et al., 2018). One previous study demonstrated that the abundance of butyrate-producing bacteria decreased significantly in the intestine of non-small cell lung cancer (NSCLC) patients (Gui et al., 2020). Interestingly, this change was directly proportional to feedback effect on the immune response in the distal lung through “lung-gut axis” (Budden et al., 2017). A 3.7-fold increase in Megasphaera and a 270-fold increase in the abundance of Clostridioides were observed in LC patients relative to the HC group. This finding demonstrates the complex and consistent dynamic change in fecal microbiome of cancer paints. A previous study showed that the abundance of Bacteroides, Veillonella and Clostridium were significantly higher in the LC patients than normal individuals (Zhang et al., 2018), consistent with our findings. LC carcinogenesis is thought to result from dysbiosis, not from activities of specific pathogens (Schwabe and Jobin, 2013).

The abundance of intestinal flora was found to be strongly associated with serum metabolic activities in the two groups. For example, LEfSe analysis revealed that the abundance of Erysipelotrichaceae_UCG_003, one of the most abundant genera in group HC group, was negatively associated with glycerophospholipid metabolism. And this negative correlation may be one of the ways involved in regulating metabolism *in vivo* and modulating tumor development. Furthermore, a strong positive correlation was found between the abundance of Clostridium and Synergistes and glycerophospholipid metabolism in patients with lung cancer, suggesting that the bacteria contribute to carcinogenic processes of lung cancer. In summary, the profile of gut flora combined with levels of serum metabolites has potential clinical significance.

Our findings notwithstanding, this study suffered several limitations. Although 16s rRNA gene sequencing is widely used for microbiota identification, it is not very effective for complete gene characterization. Also, the level of serum metabolites is influenced by several other factors such as diet (and the microbiota within it) and lifestyle. As such, identifying the source of metabolites without isotopic dietary labeling of is very challenging. Moreover, the sample size was relatively small and the data was not representative, having been collected from just a single centre. Therefore, further metagenomics and metabolomics studies utilizing larger sample sizes from multiple centers are needed to validate our findings. Even so, our findings have opened a new frontier regarding the association between gut microbiome as well as serum metabolome and cancers in general.

## Conclusion

Non-targeted metabolomics approach based on LC-MS can successfully distinguish LC patients from healthy individuals. In addition, the abundant of certain fecal microbiome such as Megasphaera, Clostridioides, Erysipelotrichaceae and Phascolarctobacterium in LC patients is significantly different from that of normal individuals. Also, the microbial diversity in LC patients is significantly higher than that of normal individuals. Particular, the serum level of certain glycerophospholipids (LysoPE 18:3, LysoPC 14:0, LysoPC 18:3) as well as AcylGlcADG 66:18;AcylGlcADG (22:6/22:6/22:6), Acylcarnitine 11:0 and Hypoxanthine can distinguish between LC patients and normal individuals. In general, the profiles of gut microbiota and serum metabolites are potential diagnostic markers for LC.

## Data Availability Statement

The datasets presented in this study can be found in online repositories. The names of the repository/repositories and accession number(s) can be found below: The BioProject database (https://www.ncbi.nlm.nih.gov/bioproject/PRJNA736821/) with accession number PRJNA736821.

## Ethics Statement

The studies involving human participants were reviewed and approved by Ethics Committee of Hangzhou First People’s Hospital. The patients/participants provided their written informed consent to participate in this study.

## Author Contributions

Conceptualization: XW and FZ. Methodology: FZ and RA. Formal analysis: FZ and RA. Data curation: FZ and RA. Software: FZ. Writing-original draft preparation: FZ and RA. Writing-review and editing: XW, FZ, RA, LW, and JS. All authors contributed to the article and approved the submitted version.

## Funding

This study was supported by Zhejiang Medical Health Science and Technology Planning Project (NO. 2019ZD014).

## Conflict of Interest

The authors declare that the research was conducted in the absence of any commercial or financial relationships that could be construed as a potential conflict of interest.

## Publisher’s Note

All claims expressed in this article are solely those of the authors and do not necessarily represent those of their affiliated organizations, or those of the publisher, the editors and the reviewers. Any product that may be evaluated in this article, or claim that may be made by its manufacturer, is not guaranteed or endorsed by the publisher.
