# Supplementary material for: Specific Gut Microbiome and Serum Metabolome Changes in Lung Cancer Patients
Source: Front Cell Infect Microbiol. 2021 Aug 30;11:725284. doi: 10.3389/fcimb.2021.725284 (PMC8435782; doi:10.3389/fcimb.2021.725284)

Supplementary table captions

Supplementary Table 1. The study cohort included clinicopathological factors of LC patients and HC volunteers.

Supplementary Table 2. Bacterial taxonomic assignment of OTUs based on exact sequence variants. HC, Healthy volunteers group; LC, Lung cancer group.

Supplementary Table 3. Heat-map analysis for the 77 discriminatory genera of bacteria by Wilcoxon rank-sum test.

Supplementary Table 4. LC-MS-based metabolite abundance data for the HC (Healthy volunteers, n = 29) and LC (Lung cancer patients, n = 27) group fecal samples. Four outlier samples (samples # 27, 29, 31, 58) were discarded. HC, Healthy volunteers group; LC, Lung cancer patient group.

Supplementary Table 5. Fold changes (FC) and Variable importance in projection (VIP) score for LC-MS-based metabolomics datasets for the HC and LC groups. Highlighted in yellow are the significantly (alpha = 0.05, p values obtained from t-test, t-test adjusted and false discovery rates (FDRs)) different metabolites, FC values highlighted in red and green represent increased and decreased metabolites in LC, and VIP score highlighted in blue is the measurement of the variable importance of metabolites.

Supplementary Table 6. List of metabolites showing higher and lower abundances in LC patients as compared to HC subjects.

Supplementary Table 7. The KEGG and HMDB metabolite IDs for the identified/quantified metabolites in the entire metabolomics study.

Supplementary Table 8. Pearson correlation (r) values for differential metabolites and differential microbes. Pearson correlation r values, p values are provided.

Supplementary figures

Supplementary Figure 1. Flow diagram of the enrollment and analysis process of the LC patients and HC volunteers.

Supplementary Figure 2. PCA (Principal Component Analysis) show that four samples (HC_33, LC_16, LC_17 and LC_18) were outliers.

Supplementary Figure 3. Relative bacterial richness and evenness analyses. (A) Good coverage represents the coverage of each sample library, and the higher the value, the higher the probability that the sequence is detected in the sample. LC, LC patient group(blue); HC, HC volunteer group(red). (B) Rarefaction curve evaluating the relative bacterial richness to determine whether further sequencing would identify additional OTUs. LC, LC patient group(blue); HC, HC volunteer group(red). (C) The Shannon index estimating the microbial diversity index in the samples. LC, LC patient group(blue); HC, HC volunteer group(red). (D) The Simpson index estimating the index of microbial diversity in the sample. LC, LC patient group(blue); HC, HC volunteer group(red). (E) The Chao1 index estimating the number of OTUs contained in the sample.LC, LC patient group(blue); HC, HC volunteer group(red). (F) Firmicutes/Bacteroidetes ratio. NS, not significant.

Supplementary Figure 4. Alterations in the composition of fecal microflora associated with LC. (A) Welch's t-test results for evaluating the relative abundance of significantly different microbiota at the phylum level. LC (yellow) and HC (blue) groups for bars and dots. (B) Welch's t-test results for evaluating the relative abundance of significantly different microbiota at the genus level. LC (yellow) and HC (blue) groups for bars and dots.

Supplementary Figure 5. Characterization of gut microbiota composition in 27 LC patients and 29 healthy controls. A Venn diagram was applied to illustrate that the gut microbiota profiling (n = 27 and n=29, LC vs HC) could represent the overall status (n = 39 and n = 40, LC vs HC) in the number and category of OTUs, phylum and differential genera.

Supplementary Figure 6. Serum metabolome of the LC and HC groups. (A) Pathway enrichment and the significance for the total metabolites identified using a LC-MS-based metabolomics platform in both groups. (B) Metabolite-metabolite (Pearson's r) correlations showing the correlation between various groups of metabolites for the combined HC and LC metabolomics datasets. High (2, red) and low (-1, green)

correlations are shown. Five discernible highly correlated groups of metabolites were (i) Lipids and lipid-like molecules, Benzene and substituted derivatives, Organic acids and derivatives, (ii) Benzenoids, (iii) Lipids and lipid-like molecules, (iv) Lipids and lipid-like molecules, Organic acids and derivatives, Organoheterocyclic compounds Benzenoids, (v)Lipids and lipid-like molecules, Organoheterocyclic compounds.

Supplementary Figure 1

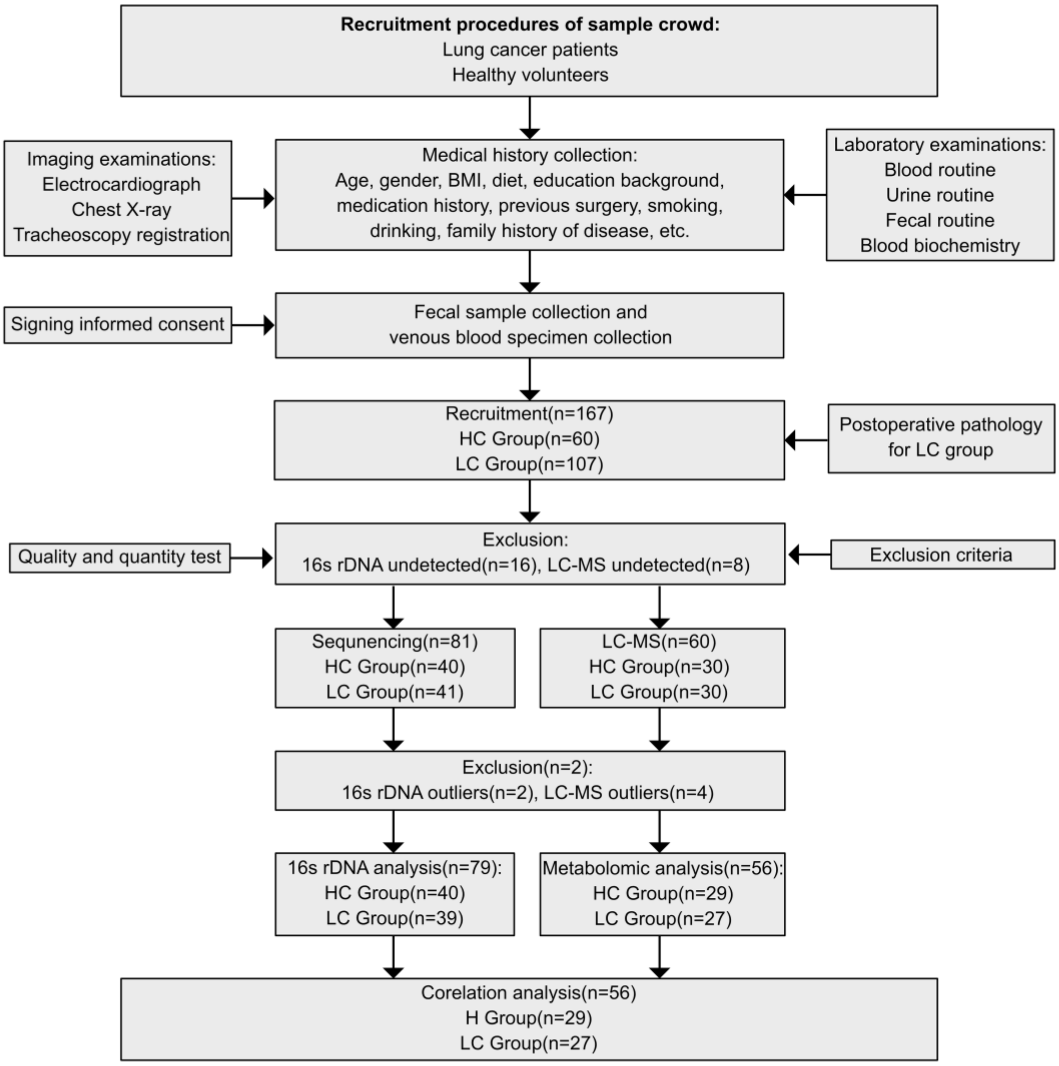

Supplementary Figure 2

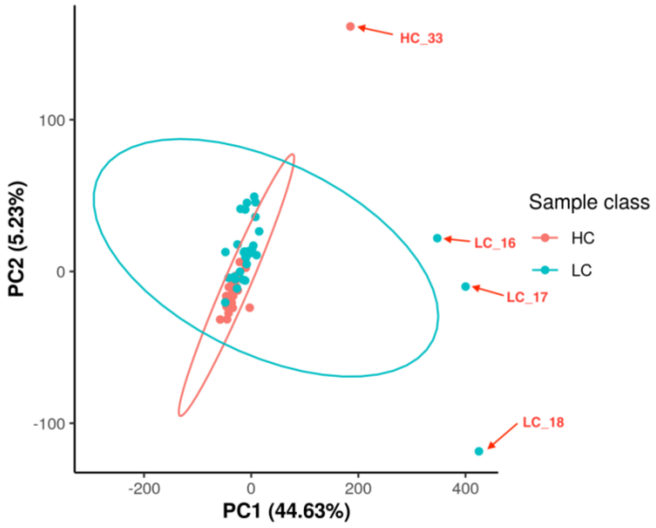

sample	PC1 (44.63	PC2 (5.23	PC3 (3.57
HC_1	-41.80716	-10.27071	13.407696
HC_10	-46.1274	-23.10473	-26.86827
HC_11	-45.02355	-31.53485	-27.88719
HC_12	-2.789317	-23.87876	-24.55739
HC_13	-41.20347	-19.47023	-8.916304
HC_14	-43.66897	-17.08002	-10.45124
HC_15	-46.55675	-16.18817	-0.567428
HC_16	-57.95234	-31.66402	-21.12129
HC_17	-45.65376	-17.50212	-9.750349
HC_18	-33.69187	-10.83207	3.1879877
HC_2	-48.41441	-20.34863	6.1173141
HC_21	-33.95944	-15.97751	-23.25529
HC_23	-33.2798	-8.662016	-3.132136
HC_24	-34.48566	-7.479125	-2.597276
HC_25	-30.27615	-8.632055	-9.633569
HC_26	-36.38162	-20.58751	-12.95196
HC_28	-35.63122	-14.70676	9.830164
HC_3	-34.40547	-23.97922	-12.72379
HC_30	-41.14285	-21.94799	-15.47664
HC_31	-10.16806	2.4119408	-27.0762
HC_32	-41.7336	-18.34739	-13.28768
HC_33	185.1747	161.40595	-87.34468
HC_34	-33.723	-16.14203	-15.99236
HC_36	-22.98861	-4.082691	-33.94463
HC_37	-42.36575	-27.44517	-29.10239
HC_38	-21.33594	6.2012086	-2.825804
HC_4	-24.98482	-12.45067	25.714979
HC_40	-27.68764	-2.618624	-37.4104
HC_5	-27.19589	-10.38923	22.011487
HC_6	-24.7472	-4.377382	30.211963

LC_1	14.864913	26.406814	-16.72867
LC_10	-31.82071	-3.518829	-9.858967
LC_11	-48.09092	12.782213	13.631351
LC_14	-20.92524	-0.224302	48.742571
LC_16	347.33725	21.93009	64.874461
LC_17	400.14029	-9.951443	20.7162
LC_18	425.07292	-118.4916	-44.56192
LC_2	-7.953179	45.204138	5.2008242
LC_20	-29.88516	-5.080718	42.436083
LC_21	-48.41141	-20.41512	19.275441
LC_22	-26.45102	-11.20238	-16.75768
LC_23	-5.697946	13.309351	26.820061
LC_24	-42.20577	-4.175213	9.8057661
LC_25	-8.402591	4.8796288	-15.74104
LC_26	1.5527116	14.728189	1.7439237
LC_27	3.9006945	17.010285	72.42105
LC_28	-11.28213	-5.975524	6.5044337
LC_29	-15.28405	-5.443148	-10.17656
LC_30	-25.591	17.779086	46.85003
LC_31	-12.80838	13.12167	17.07587
LC_32	-9.527957	9.1638394	3.1114832
LC_33	-10.57916	9.5618785	-17.48601
LC_36	-0.726834	12.130135	37.281102
LC_37	-13.61958	12.595775	34.310378
LC_38	10.021945	10.737877	23.561477
LC_4	8.0191994	45.59337	-13.56119
LC_5	7.9104534	35.927454	-5.006405
LC_6	-19.91758	41.22669	-8.713497
LC_7	-11.07003	40.801274	-7.582939
LC_8	5.6372795	49.269115	18.205046

Supplementary Figure 3

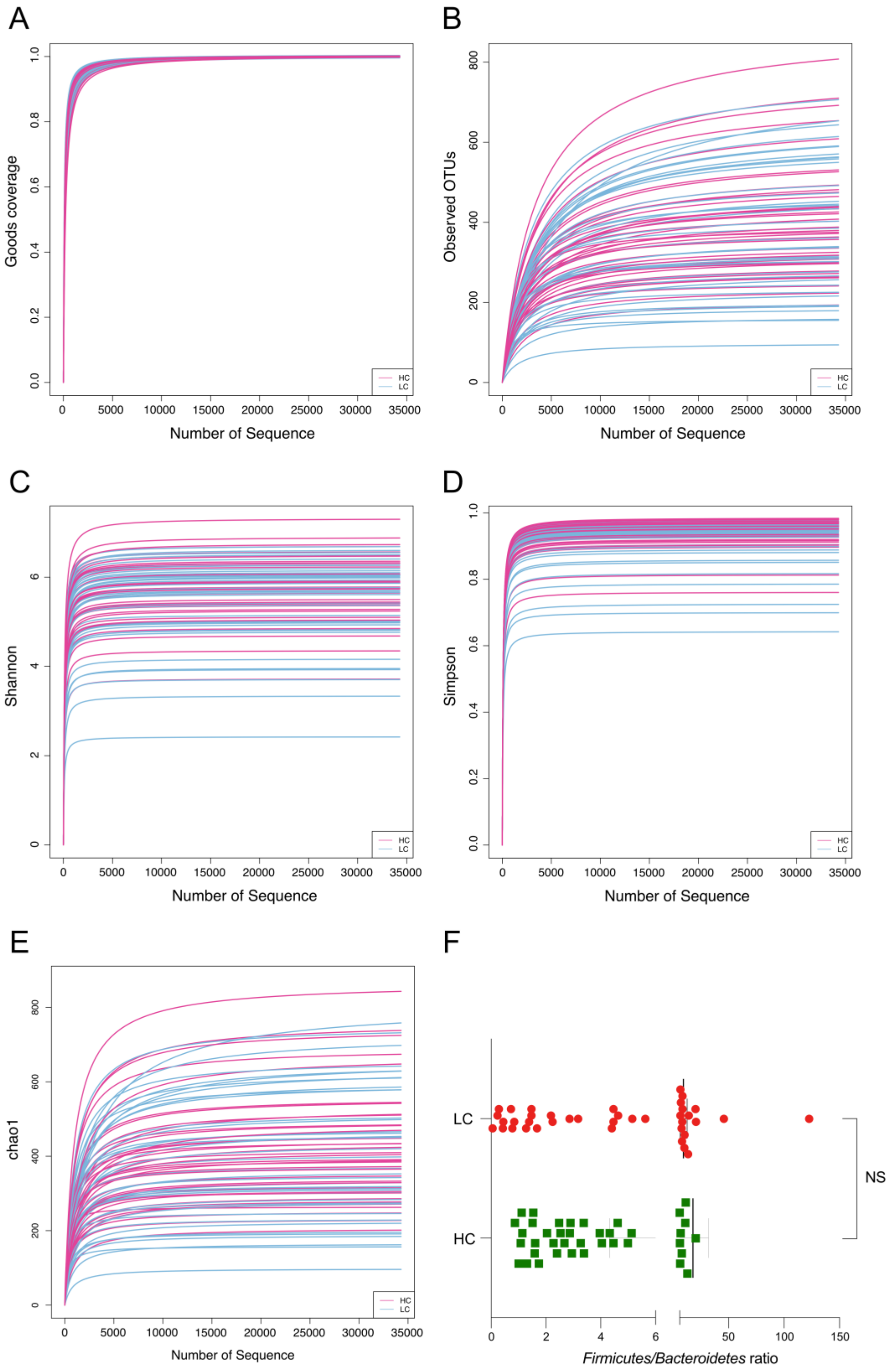

Supplementary Figure 4

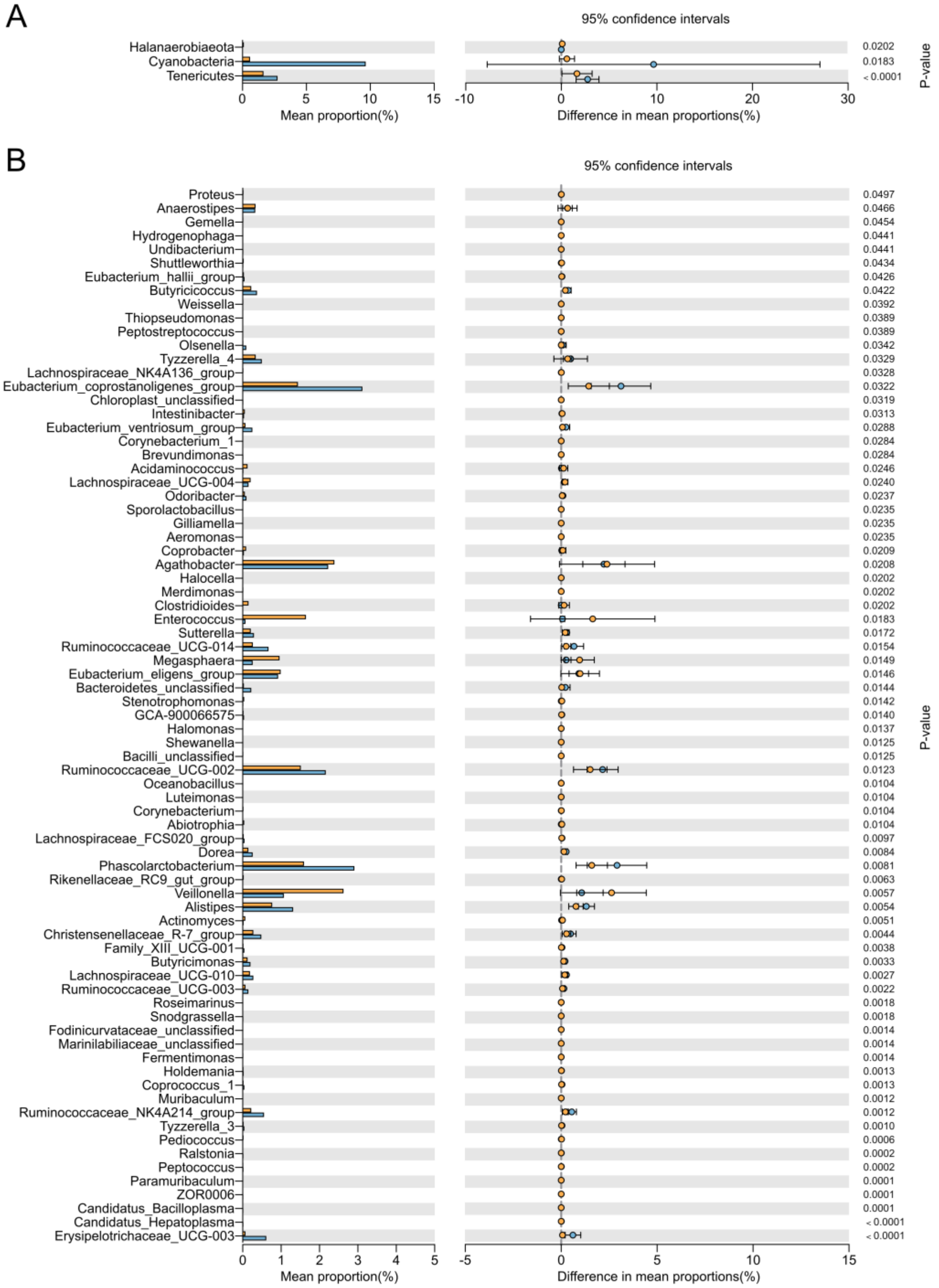

Supplementary Figure 5

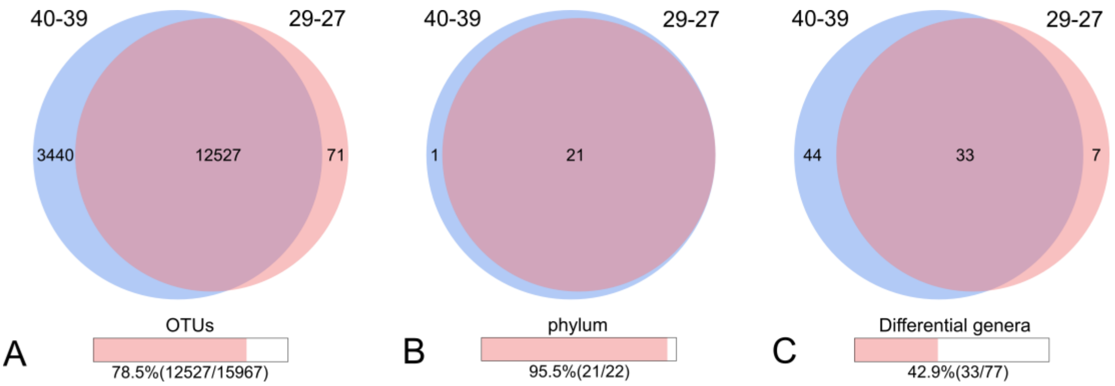

Supplementary Figure 6

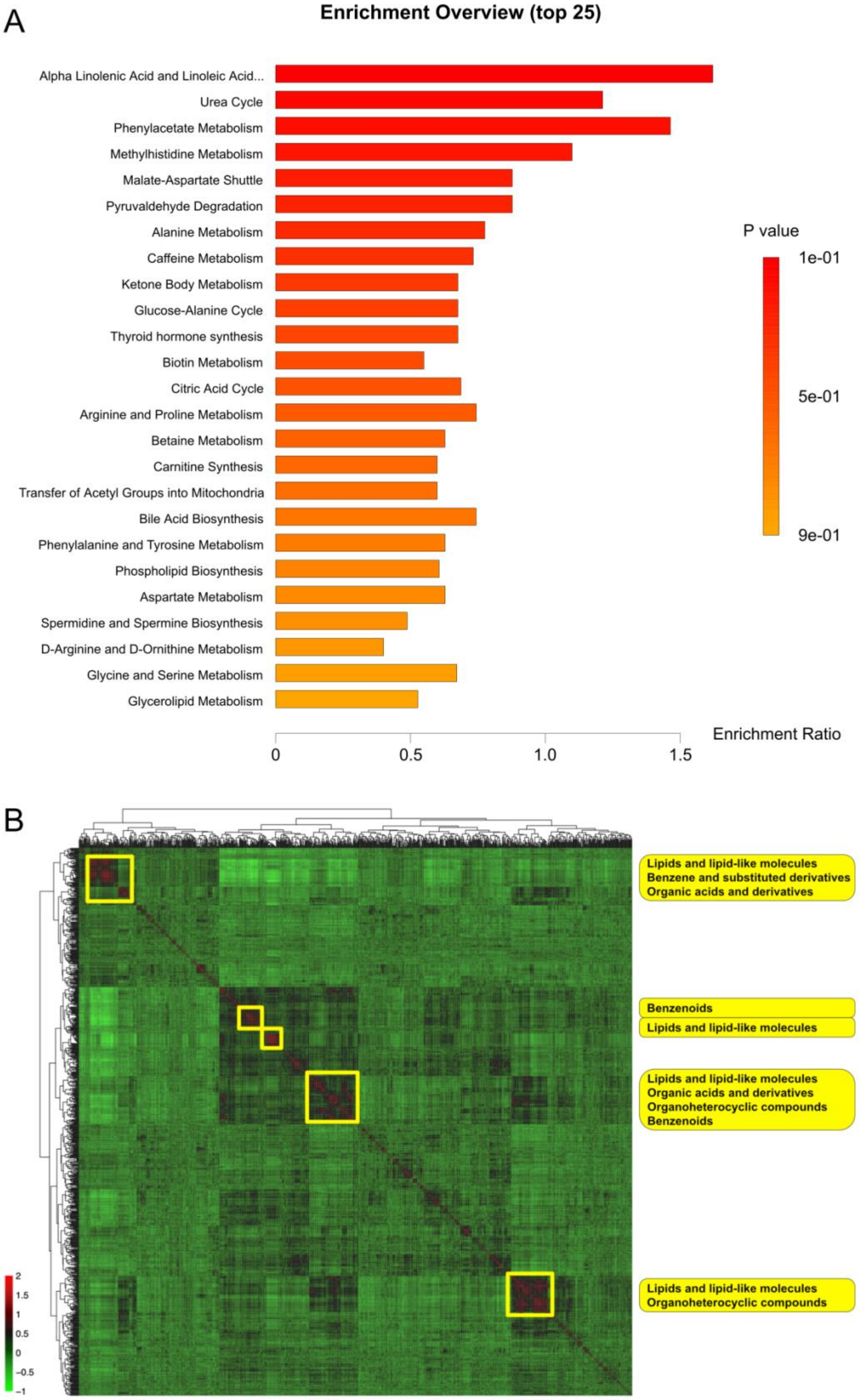

Supplement: Supplementary file 1 [file DataSheet_2.pdf]
